# Force-Moment Sensor for Prosthesis Structural Load Measurement

**DOI:** 10.3390/s23020938

**Published:** 2023-01-13

**Authors:** Md Rejwanul Haque, Greg Berkeley, Xiangrong Shen

**Affiliations:** Department of Mechanical Engineering, The University of Alabama, Tuscaloosa, AL 35401, USA

**Keywords:** force-moment sensor, loadcell, prosthesis load measurement

## Abstract

Measurement of prosthesis structural load, as an important way to quantify the interaction of the amputee user with the environment, may serve important purposes in the control of smart lower-limb prosthetic devices. However, the majority of existing force sensors used in protheses are developed based on strain measurement and thus may suffer from multiple issues such as weak signals and signal drifting. To address these limitations, this paper presents a novel Force-Moment Prosthesis Load Sensor (FM-PLS) to measure the axial force and bending moment in the structure of a lower-limb prosthesis. Unlike strain gauge-based force sensors, the FM-PLS is developed based on the magnetic sensing of small (millimeter-scale) deflection of an elastic element, and it may provide stronger signals that are more robust against interferences and drifting since such physical deflection is several orders of magnitude greater than the strain of a typical load-bearing structure. The design of the sensor incorporates uniquely curved supporting surfaces such that the measurement is sensitive to light load but the sensor structure is robust enough to withstand heavy load without damage. To validate the sensor performance, benchtop testing of the FM-PLS and walking experiments of a FM-PLS-embedded robotic lower-limb prosthesis were conducted. Benchtop testing results displayed good linearity and a good match to the numerical simulation results. Results from the prosthesis walking experiments showed that the sensor signals can be used to detect important gaits events such as heel strike and toe-off, facilitating the reliable motion control of lower-limb prostheses.

## 1. Introduction

Human walking is a cyclic movement that involves coordinated reciprocating motion of two legs. Despite the continuous forward motion of the upper body, each leg’s motion pattern is essentially discrete, with vastly different dynamic behaviors across different phases in a single gait cycle [1]. During the swing phase, the leg joints (especially the knee) display low impedance, generating a pendulum-like swing motion to reposition the leg in front of the upper body to prepare for the next heel strike. During the stance phase, the leg joints display much elevated impedance, preventing the leg from buckling under the load imposed by the body weight. For individuals living with lower-limb losses, restoring the locomotive functions require their prosthetic devices to replicate the dynamic characteristics of the biological legs, especially the significant differences between the stance and swing phases.

Leg prostheses, in the early stage of prosthetics development, was simple, purely mechanical devices with very limited capability of regulating joint behavior. With the technological advances in actuation, electronics, and control, micro-processor-controlled (MPC) knee prostheses were developed and entered clinical practice, for example, Ottobock C-Leg [2] and Ossur Rheo Knee [3]. Typical MPC prostheses regulate joint-resistance during different gait phases with hydraulic or pneumatic circuits, and some also provide swing assistance using mechanical springs [4]. Unlike the control of upper-limb prostheses [5], realizing such functionality in leg prostheses requires accurate and reliable detection of gait phases in real-time, which is usually conducted through the measurement of prosthesis structural load (e.g., using a sensorized pylon in the C-Leg). More recently, powered robotic lower-limb prostheses started to emerge, including the Vanderbilt Leg [6,7], MIT ankle-foot prostheses [8], Ossur PowerKnee [9], Open Source Leg [10], and Utah Leg [11]. With their capability of providing actively-powered joint action, the importance of prosthesis structural load is further highlighted, not only for the determination of the gait phase, but also for the quantification of the prosthetic leg load-bearing for more sophisticated functions (e.g., to trigger the sit-to-stand assistance when an amputee shifts load to his/her leg prosthesis [12]).

For load measurement, the most extensively used method is strain measurement. Strain gauges can be firmly attached to the load-bearing structure to measure the underlying micro-scale deflection, which correlates to the magnitude to the applied load [13]. Strain gauge-based load measurement has been applied to orthopedics since the 1970s [14], and it still remains as a major approach for the prosthesis structural load measurement. Strain gauges can be attached to structural components (e.g., pylons), or a dedicated structure can be designed and instrumented to form a force sensor (i.e., load cell). For example, Sup et al. developed a 3-axis socket load cell for the control of an early version of the Vanderbilt Leg in which regions of compression and tension were defined in a double-cross structure for strain measurement [6]. Despite the maturity of such a technique, the weak signals generated by strain gauges are susceptible to interferences, and signal drift may also occur and affect the consistency of the measurement. An alternative approach, similar to the strain gauge method, is the force-sensing resistor (FSR)-based load sensing [15]. FSRs can be placed under the prosthetic feet [16,17] or embedded in the prosthesis structure [18] for load sensing, but due to their unsatisfactory measurement accuracy and consistency, FSR signals use has largely been limited to the detection of gait events (e.g., heel strike) for phase transition.

The most recent progress in the area was the structural deflection-based prosthesis load sensing. Instead of using strain gauges to measure micro-scale deflection, this new sensing approach is based on the measurement of much larger (typically millimeter-scale) deflection of load-bearing structures. A typical example is the instrumented pyramid adapter developed by Gabert and Lenzi [19], which incorporates a cantilever beam-type structure as the load-sensing element. By using two magnetic sensors to measure the displacements of the two ends of the cantilever beam, this new sensor is able to provide robust sensing of the axial force and bending moment in the sagittal plane. However, the cantilever beam structure needs to be accurately machined with high-performance metal (titanium in the prototype), resulting in a high cost of manufacturing. Another example is the parallelogram load cell developed for the Vanderbilt Leg [7], which also provides reliable load measurement through a parallelogram-configured deformable structure. As its major weaknesses, this load cell only measures the axial force (not the bending moment), and it is relatively heavy due to the use of a coil spring as the elastic element.

Motivated by the weaknesses of existing prosthesis load sensors and sensing approaches, we developed a novel prosthesis load sensor, namely Force-Moment Prosthesis Load Sensor (FM-PLS), to provide reliable measurement of the axial force and bending moment in a lower-limb prosthesis with a simple, lightweight, and inexpensive sensor package. Compared with the strain gauge-based load sensors, the FM-PLS provides stronger signals, more robust against interferences. Compared with the recent large structural deformation-based sensors, the FM-PLS is mechanically simpler and less expensive to manufacture. Specifically, compared with the recent work by Gabert and Lenzi [19], the FM-PLS uses a dedicated elastic element to measure the load (axial force and bending moment) through its deflection, which is easy and less expensive to manufacture since no high-accuracy machining is needed. Further, with the use of the curved supporting surfaces of the mounting blocks (upper and lower), the deflection of the elastic member can be modulated such that the sensor has high sensitivity when light load is applied while still being able to endure heavy load without damage.

This paper details the development of this FM-PLS sensor along with the related results obtained through finite-element analysis and experimental testing. The paper is organized as follows: Section 2 presents the overview of the FM-PLS design and development, including sensing principle and electronic components; Section 3 presents the experimental procedure while Section 4 presents the results to validate the performance of the FM-PLS; Section 5 presents the discussion; and Section 6 summarizes the conclusions.

## 2. Design and Development of the FM-PLS

### 2.1. Functioning Mechanism of FM-PLS

The functioning mechanism of the FM-PLS is based on two-stage transduction: (1) Displacement due the deformation of elastic element by applied force or bending moment, and (2) Magnetic field transduction due to change in distance between the magnet and magnetic field transducer.

**Figure 1:**
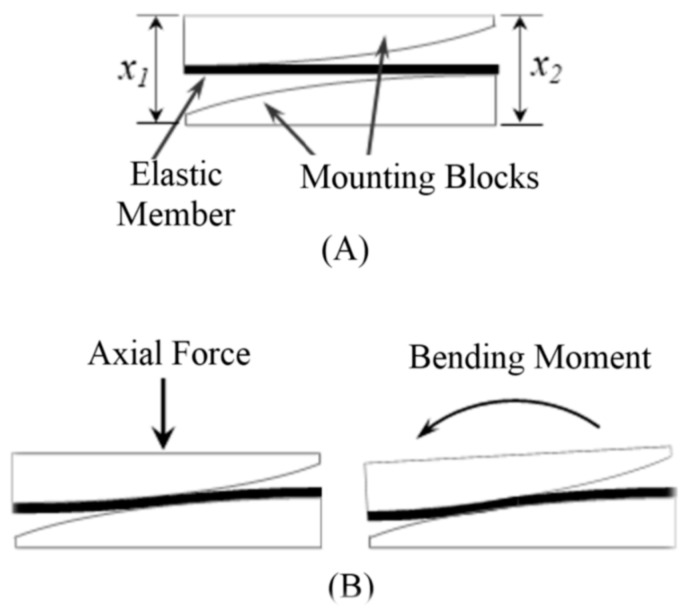
Functioning mechanism of the FM-PLS: (**A**) schematic of the sensor structure, and (**B**) deflections under axial force and bending moment.

The key component of 1st-stage transduction is a flat carbon fiber or fiberglass piece, which serves as the elastic element to deform under load. Each end of this elastic element is rigidly attached to a curved mounting block in a cantilever beam-like configuration, as shown in Figure 1. Under an axial force, the left and right ends experience symmetric deflections (i.e., ∆x_1_ = ∆x_2_); under a bending moment, the left and right ends experience asymmetric deflections (i.e., ∆x_1_ ≠ ∆x_2_) (Figure 1B). As such, any combination of the axial force and bending moment can be measured by independently measuring the small deflections at the left and right ends (∆x_1_ and ∆x_2_).

The deflection of the elastic member changes the relative distance between the magnets and the magnetic field sensors located on top/bottom of the magnets. Since the magnetic flux density is a function of distance from the magnet, magnetic flux density measured by the magnetic field transducers changes due to the change of distance by applying force. The output of the magnetic field transducer is voltage. Thus, the applied force is sensed as the voltage by the transducer through two-stage transductions.

Both stages of the transduction principle have a substantial impact on its capacity to measure force and bending moment. To establish the relationship between applied force and magnetic field sensor voltage output, analyses (either simulation or mathematical) have been carried out in multiple stages. The elastic member between the upper and lower blocks works as a cantilever beam. Hence the deflection of the elastic member is a function of the active length of the cantilever beam. This sensor design adopts a curved surface for both upper and lower blocks (as shown in Figure 1) which modulates the active length of the elastic member and thus controls the deflection. That means this feature makes the sensor sensitive at low force while still withstanding heavy load without damage.

**Figure 2:**
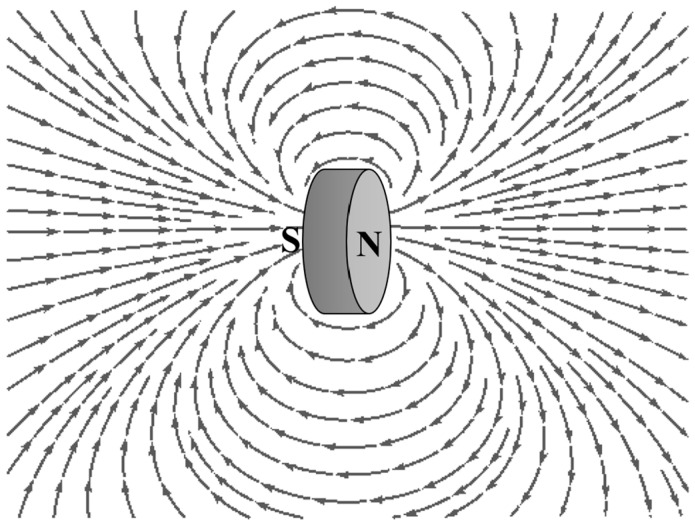
Magnetic field distribution of a cylindrical bar magnet.

The second stage of transduction relies on the amount of magnetic flux measured by the magnetic field sensor. Although the magnetic field is related to the distance from the magnet, the magnetic fields around a magnet are not uniform in all directions. The magnetic field depends on numerous factors including the shape of the magnet, the distance between the poles, as well as the specific position around the magnet. The magnet used in the sensor design can be considered as the cylindrical bar magnet whose two opposite faces are the two poles. The magnetic field distribution around a cylindrical bar magnet [20] can be shown in the Figure 2.

**Figure 3:**
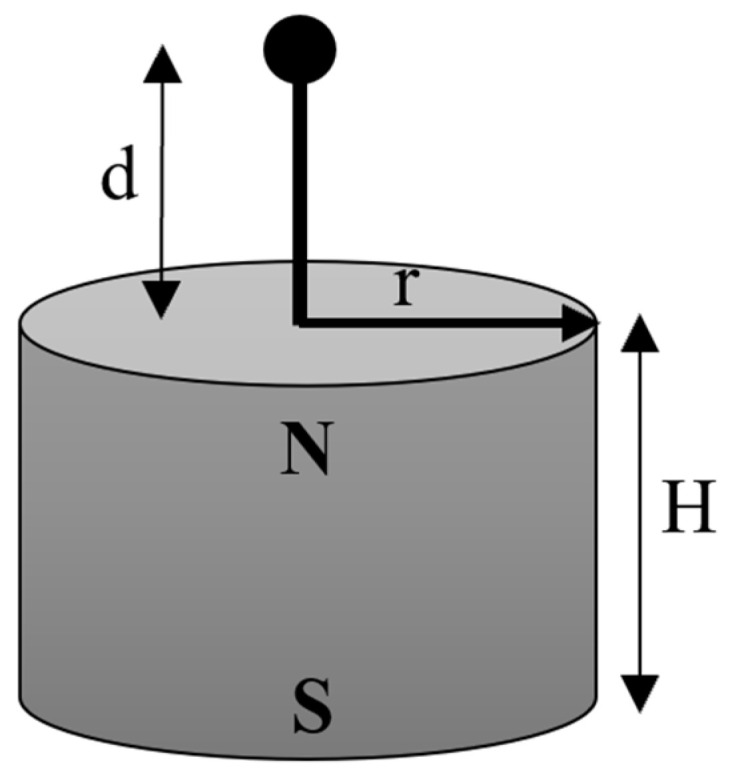
The magnetic flux density of a cylindrical bar magnet (radius r and thickness H) in the axial direction at a distance d.

The magnetic flux density at a distance d (as shown in Figure 3), in the axial direction, can be expressed as Equation (1) [21], where H is the thickness of the magnet, r is the radius of the magnet and B_r_ is the radial component of the magnetic flux density in the axial direction.

(1) $$B=\frac{B_{r}}{2}\left(\frac{H+d}{\sqrt{r^{2}+{\left(H+d\right)}^{2}}}-\frac{d}{\sqrt{r^{2}+d^{2}}}\right)$$

### 2.2. Overview of the Design

The main design objective of the FM-PLS was to measure the axial force and bending moment simultaneously while minimizing the mechanical complexity.

**Figure 4:**
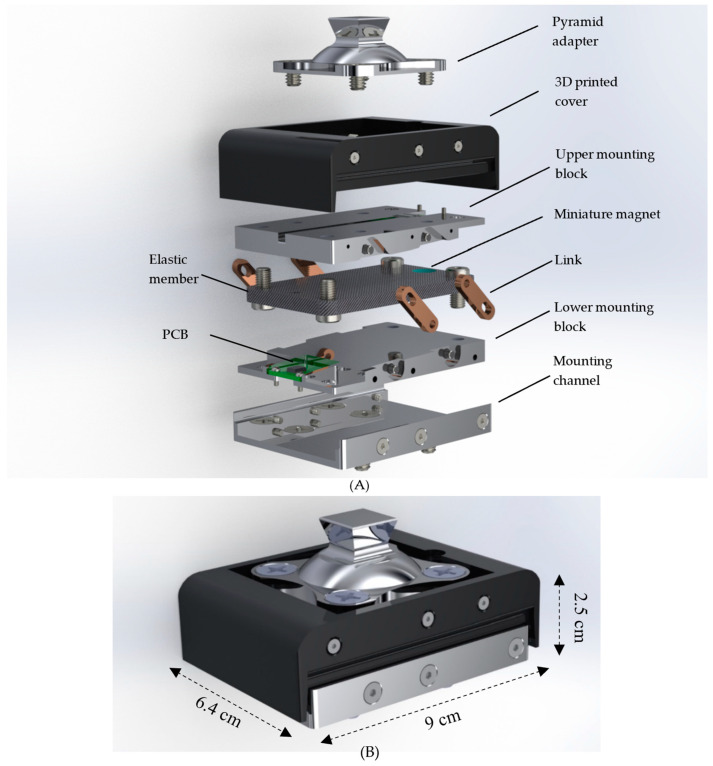
Detail design of the FM-PLS sensor: (**A**) Exploded view, and (**B**) FM-PLS sensor with dimensions.

The FM-PLS consists of four main structural components and two sets of sensing components. The structural components are: (1) Elastic member, (2) Upper mounting block, (3) Lower mounting block, and (4) Detachable adapter, as shown in Figure 4. The elastic member is the sensor’s core structural component which is mounted to the upper and lower block on each end. By measuring the upper and lower blocks relative displacement (determined by the elastic deflection of the member), the axial force and bending moment can be determined accordingly. The mating surface of the lower/upper block with the elastic member is curved to modulate the deflection of the elastic member.

The sensing components consist of magnetic field sensors and magnets. For the measurement of the deflections, a miniature magnet is embedded on each end of the elastic element, and a magnetic sensor is embedded in the free end of each curved mounting block, measuring the change of the magnetic field due to the movement of the corresponding magnet. As such, the magnet sensors’ signals serve as indirect measurements of the force/moment applied to the sensor.

Note that each magnetic sensor is embedded in a small, printed circuit board (PCB), which also includes the signal conditioning circuit. As such, the output signals of the FM-PLS can be directly routed to the prosthesis microcontroller as a plug-n-play component.

**Figure 5:**
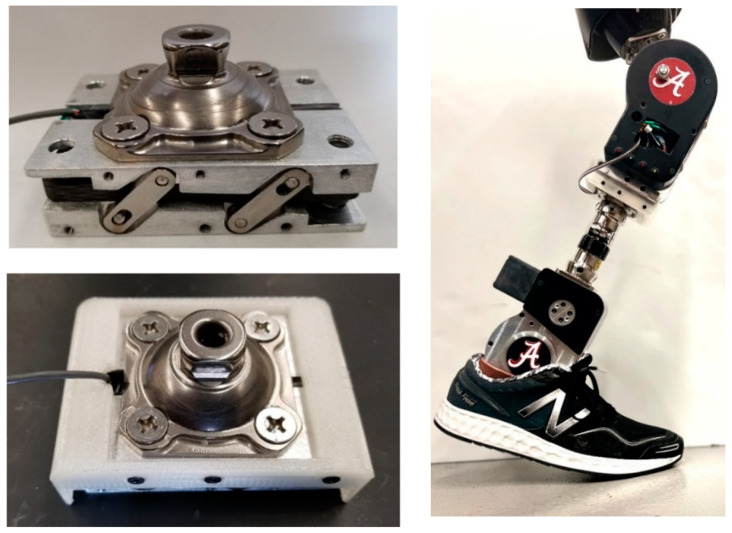
Prototype of the FM-PLS sensor (**left**) and FM-PLS fitted on a robotic prosthesis.

Further, the standard pyramid connector is mounted to the top of the FM-PLS, and a detachable mounting channel is added at the bottom of the sensor to allow the sensor to be mounted to a lower-limb prosthesis using the threaded holes for the standard pyramid connector (i.e., attach the mounting channel to the prosthesis using the existing mounting holes for the pyramid connector; subsequently, attach the rest of the sensor to the mounting channel; finally, mount the pyramid connector to the top of the sensor). As such, the FM-PLS can be easily integrated into the prosthesis structure without affecting the standard connection interface, essentially forming a sensorized pyramid connector (similar to [19]). To facilitate the sensor’s practical use in lower-limb prostheses, a pair of slotted bars are added on each side to limit the extension when a pulling force is applied (e.g., during the swing). The elastic member is made of E-fiberglass/epoxy with 67% fiber volume ratio (GC-67-UB, PolyOne Corporation, Ohio, USA) while the rest of the structural components are made of 7075 aluminum. Finally, a 3D-printed cover (made with PLA Plastic) is added to encapsulate the entire sensor and protect the interior components for its future practical use. The 3D dimensions of the FM-PLS sensor are 90 mm × 64 mm × 25 mm (without the pyramid adapter), as shown in the Figure 4B. The prototype of the FM-PLS sensor is shown in Figure 5.

### 2.3. Electrical Components

**Figure 6:**
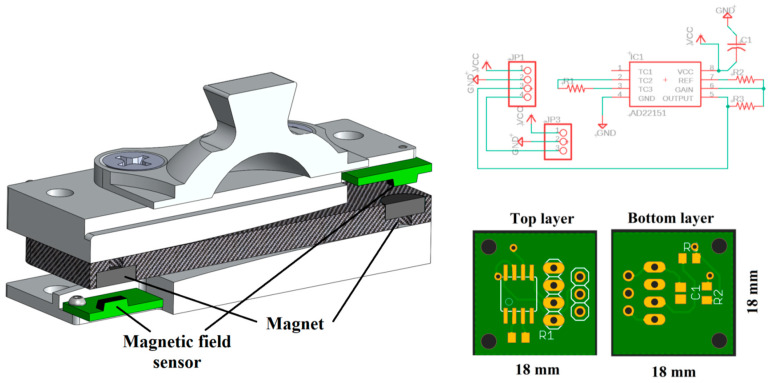
Section view of the FM-PLS showing placement of electrical component and magnet (**left**); The electrical circuit diagram (**top right**) and custom-printed circuit board (**bottom right**).

A custom-printed circuit board (PCB) was designed and developed to house the magnetic field sensor along with other electronic components necessary for the force/moment transduction and signal conditioning. The AD22151 (Analog Devices, Inc., Wilmington, MA, USA) was used as the magnetic field sensor. The schematic diagram of the PCB is shown in Figure 6 (top right) while in Figure 6 (bottom right) shows the PCB layout. Two identical, small PCBs (18 mm × 18 mm) were made for each side of the FM-PLS. The PCBs are mounted on the upper and lower block such that the AD22151 sensor stays on top/bottom of the magnet. Both boards are connected by electrical wires to share the power supply as well as to facilitate the access of both sensor signals from one PCB. The sensor signal can be read in real-time using Analog-to-Digital Converter (ADC) interface.

### 2.4. Finite Element Analysis

To determine the structural displacement, Finite Element Analysis (FEA) was conducted using Ansys Mechanical (Ansys Inc., Canonsburg, PA, USA) under various loading conditions. Only the elastic member and upper/lower mounting blocks were considered as they are the primary contributors of the displacement study. The analysis was further simplified by considering both blocks to be rigid, as they are connected to other structural components of the prosthesis and thus the deflections are negligible compared to that of the elastic member. The elastic member was composed of 33,299 quadratic tetrahedrals and material properties for E-fiberglass/epoxy with 67% fiber volume ratio (GC-67-UB, PolyOne Corporation, Avon Lake, OH, USA) were assigned and summarized in Table 1. With the symmetry of the assembly, only half of the components in their longitudinal direction were considered and symmetric boundary conditions were applied to the appropriate surfaces. The mounting blocks were constrained to the elastic member by line body connections representing the low alloy steel fasteners of the assembly. Finally, the adjoining surfaces of the elastic member to the respective mounting blocks were allowed to freely slide in contact but penetration was penalized to achieve accurate deformations of the assembly.

**Figure 7:**
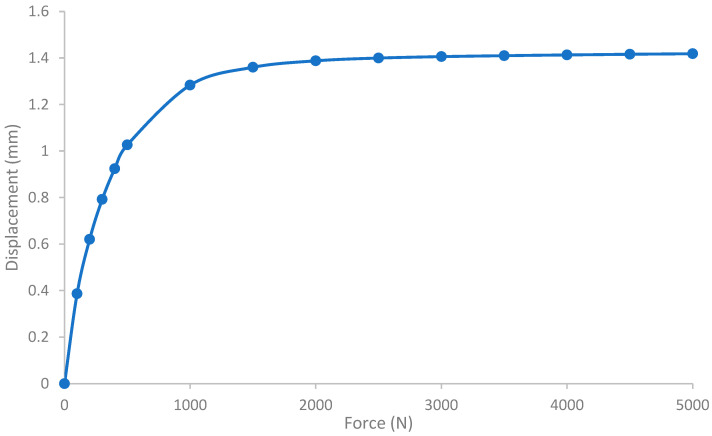
FEA result: displacement of the elastic element under axial load. The displacement has been calculated at the location of the magnet.

**Figure 8:**
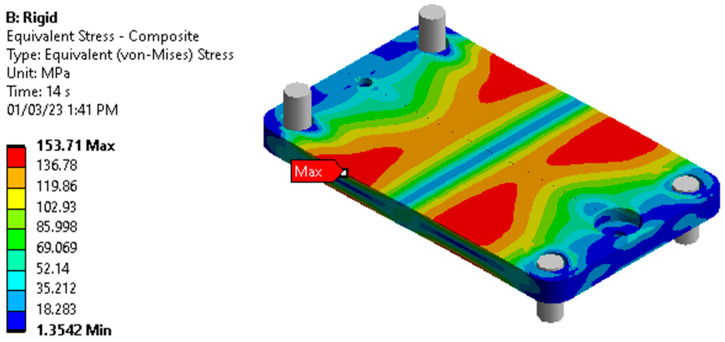
FEA: stress on the elastic element by applying 5000 N axial load.

The maximum force was chosen as 5000 N in the analysis which is more than six times of average human adult weight. Figure 7 the displacement of the elastic member at the location of the magnet by varying the axial force from 0 N to 5000 N. This was achieved over 14 linearly incremented steps in a quasistatic fashion. The figure shows that the trend of the displacement is nonlinear as the load increases; rather the displacement gradient is decreasing as the load increases. The von mises stress of the FEA is shown in Figure 8. The maximum stress on the elastic member is 153.71 MPa, which is lower than the tensile strength of the elastic member, meaning it withstands heavy load without damage.

## 3. Experimental Validation

### 3.1. Benchtop Testing

**Figure 9:**
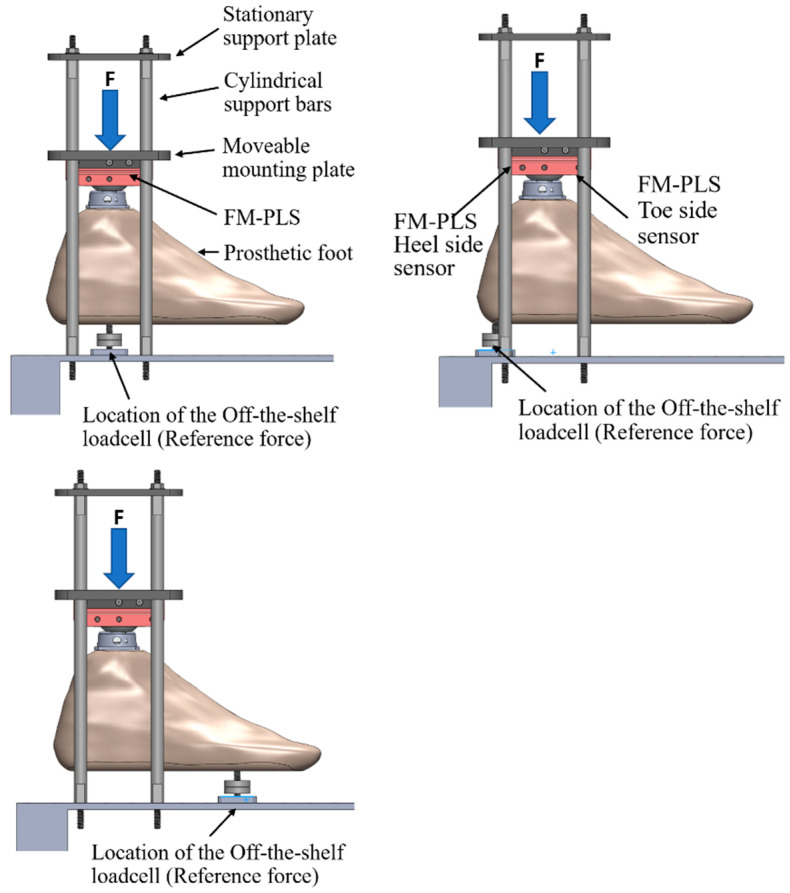
FM-PLS benchtop testing setup.

The goal of the benchtop testing was to monitor the loading response of the FM-PLS using an off-the-shelf loadcell as a reference measure. A testing setup was custom designed and fabricated for the benchtop testing of the FM-PLS in different loading conditions. The setup consists of a stationary support plate, moveable mounting plate, and four cylindrical support bars, as shown in Figure 9. An off-the-shelf prosthetic foot is mounted to the movable mounting plate through the prosthetic load sensor to be tested (FM-PLS in the experiments). The moveable mounting plate with four through-holes (with sleeve bearings installed), slides along four cylindrical support bars (serving as vertical sliding rails). This ensures the prosthetic foot can move upwards or downwards while remaining horizontal. To apply a pushing force, a lead screw assembly is used to expand the distance between the stationary support plate and the moveable mounting plate. To provide the ground truth measurement of the applied load (any combination of the axial force and bending moment), an off-the-shelf load cell (ELPF-500-T3E, Measurement Specialties, Hampton, VA, USA) supports the prosthetic foot from underneath at a selected position corresponding to the desired loading condition. In this experiment, the loadcell was positioned at three locations underneath the foot piece, which are: (1) aligned with center of the FM-PLS, (2) at the heel of the prosthetic foot, and (3) at the toe (ball joint) of the prosthetic foot, as shown in Figure 9.

### 3.2. Testing with Prosthesis

The FM-PLS was fitted with a prototype knee prosthesis to evaluate its performance. FM-PLS was interfaced with the prosthesis main controller unit to feed the sensor signals to the controller. The prosthesis controller uses finite-state impedance control strategy, for which the real-time detection of important gait events is crucial. FM-PLS signals will be used in this experiment to identify those important gait events for the controller.

**Figure 10:**
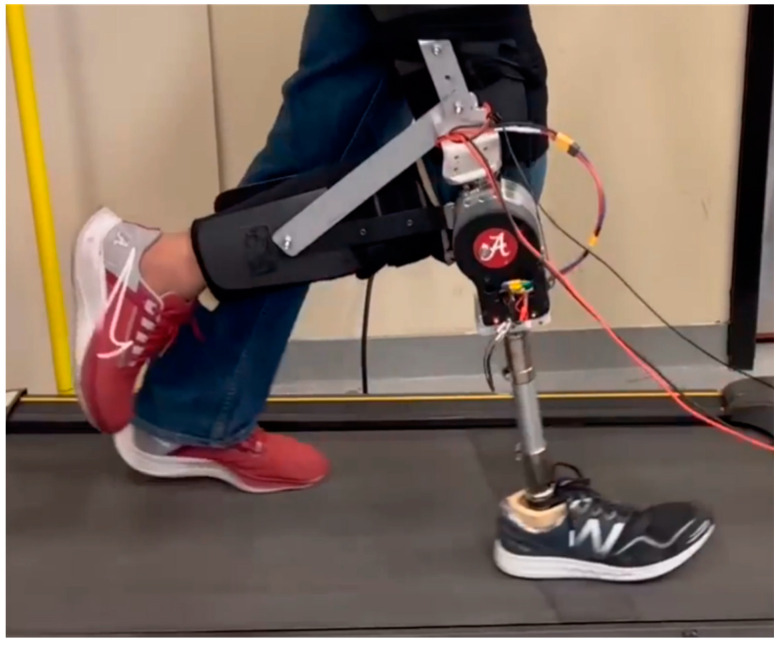
Prosthesis fitted with the FM-PLS sensor prototype.

An able-bodied male participant (31 years old, 175 cm, and 70 kg) participated in this study. An able-bodied prosthesis testing-adapter was used to attach the prosthesis to the subject. The experimental setup is shown in Figure 10. The participant walked on a treadmill at two different speeds (1 mph and 2 mph) while a total of 94 walking cycles were recorded. The duration of the experiment was 338 s and a total of 173,160 samples were taken.

## 4. Results

### 4.1. Benchtop Testing vs. Numerical Simulation

**Figure 11:**
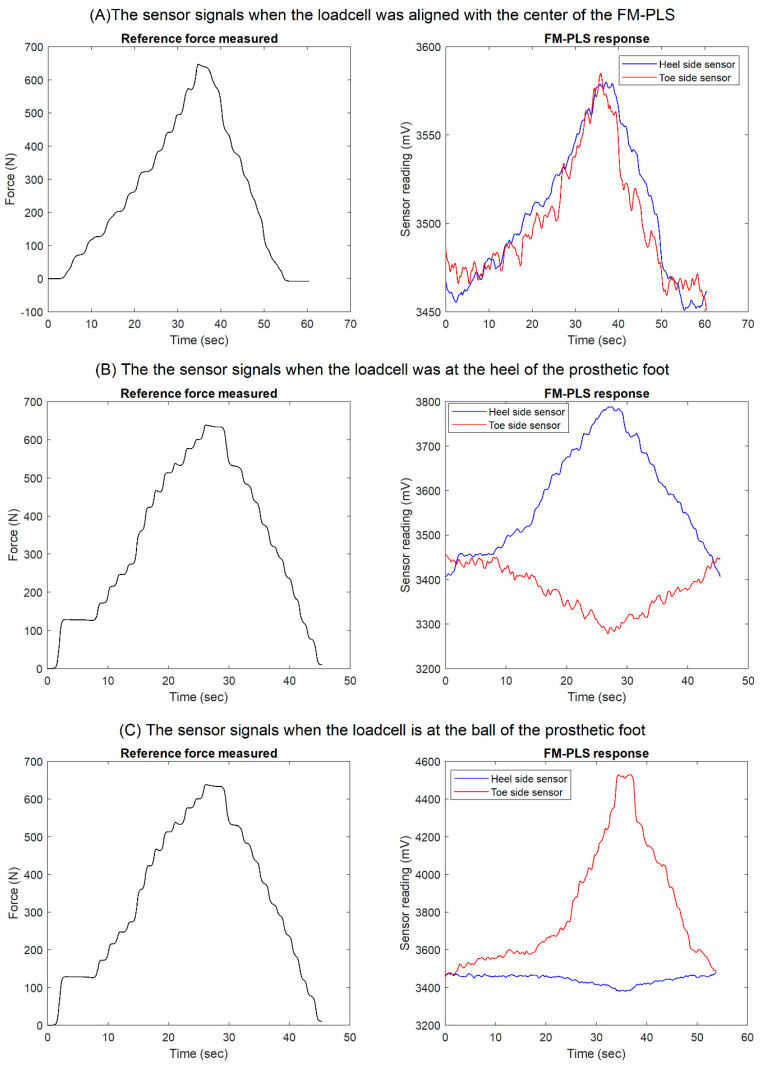
Benchtop testing results: reference measure, heel side and toe side magnetic flux sensor responses while the off-the-shelf loadcell was (**A**) aligned with center of the FM-PLS (shown in Figure 9, left column), (**B**) at the heel of the prosthetic foot (shown in Figure 9, middle column), and (**C**) at the ball of the prosthetic foot (shown in Figure 9, right column).

The results of the benchtop testing while placing the off-the-shelf loadcell underneath of the prosthetic foot as a reference measure are shown in Figure 11. Both heel side and toe side magnetic flux sensor responses are depicted while the off-the-shelf loadcell was (1) aligned with center of the FM-PLS (shown in Figure 9, left column), (2) at the heel of the prosthetic foot (shown in Figure 9, middle column), and (3) at the ball of the prosthetic foot (shown in Figure 9, right column).

**Figure 12:**
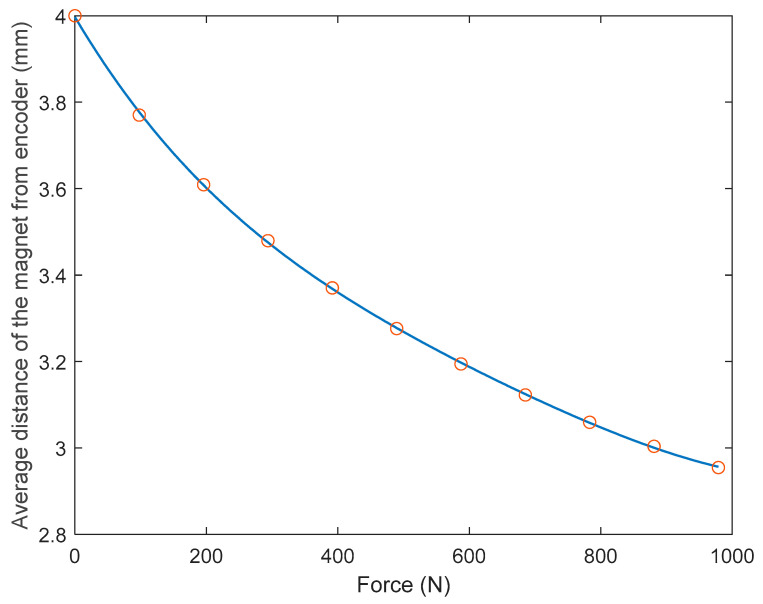
The change of relative distance between the magnet and the magnetic field transducer with respect to the change of axial force and their 4th-order polynomial fitting.

Based on the FEA, Figure 12 shows the change of relative distance between the magnet and the magnetic field transducer with respect to the change of axial force, along with their 4th-order polynomial fitting. As shown in this figure, the 4th-order polynomial model can express the force-distance relationship with good accuracy. The coefficients of the 4th-order polynomial model are as follows: 4.8069× 10^−10^, −2.8442× 10^−7^ 6.9600× 10^−5^, −0.01139, and 3.9971.

**Figure 13:**
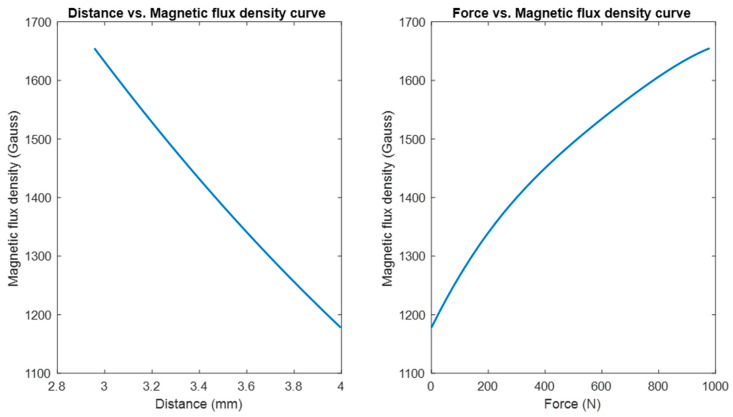
The change of magnetic flux density with respect to the relative distance between the magnet and the magnetic field transducer (**left**). The change of magnetic flux density with respect to axial force (**right**).

Figure 13 (left) illustrates the change in magnetic flux density with respect to the relative axial distance between the magnet and the magnetic flux transducer. By combining the force-distance relationship expressed by the 4th-order polynomial model and the distance-magnetic flux density relationship given by Equation no. (1), the force-magnetic field relation was established, as shown in Figure 13 (right). Since the voltage output of the magnetic flux density transducer is proportional to the magnetic field, the force-magnetic field model can be converted to the force-voltage output model.

**Figure 14:**
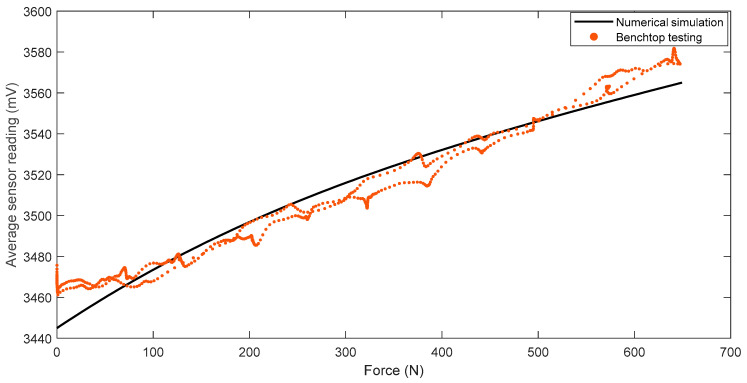
Comparison of sensor readings from numerical simulation and benchtop testing of the FM-PLS during the axial force change.

When the off-the-shelf loadcell is positioned at the center, the applied load on the FM-PLS can be considered as the axial force. Figure 14 shows the experimentally measured FM-PLS sensor readings while the axial force was gradually increased from 0 to 650 N. The figure also shows the simulated sensor reading (based on numerical simulation) for the similar axial force.

**Figure 15:**
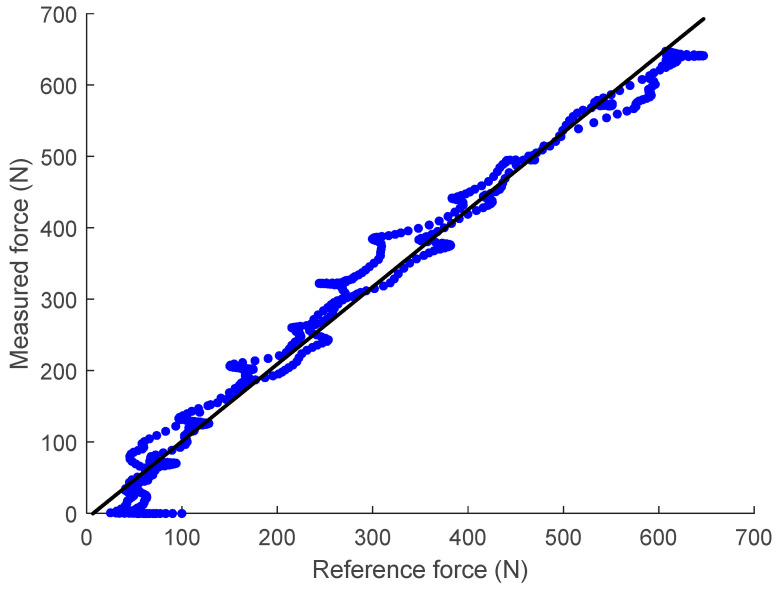
Linear regression of the unfiltered data taken from the FM-PLS sensor and reference off-the-shelf loadcell.

Figure 15 shows the scatter plot of the reference force (measured by the off-the-shelf loadcell) versus the FM-PLS signals in the benchtop testing, along with the linear regression fitting. The fitting has an R-square value of 0.9802 and a root-mean-squared-error (RMSE) of 28.59 N.

### 4.2. Testing with Prosthesis

**Figure 16:**
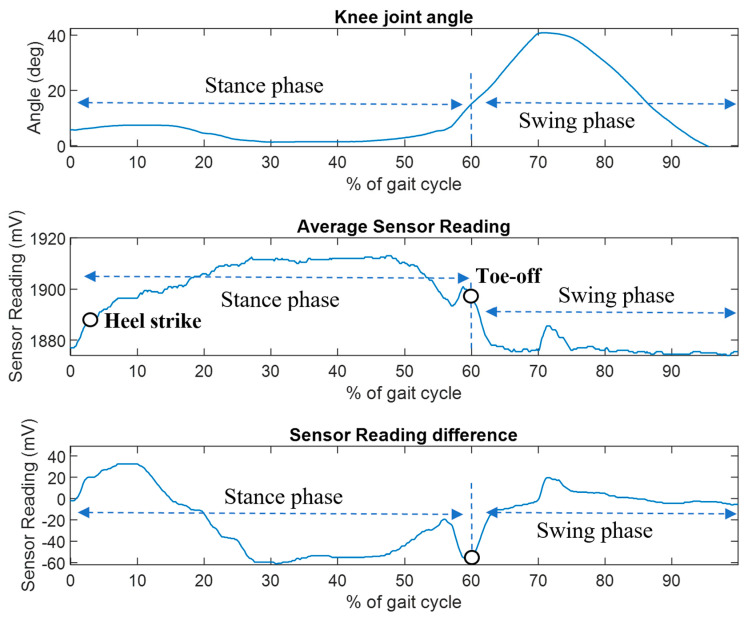
FM-PLS walking test: knee angle (**top figure**), average sensor reading (**middle figure**), and sensor reading difference (**bottom figure**).

The results of the prosthesis treadmill walking experiments are shown in Figure 16, which shows the FM-PLS sensor response during a complete gait cycle along with the relative knee angle measured from the knee prosthesis encoder. A complete walking gait cycle consists of the stance phase (~60% of the cycle) and the swing phase (~40% of the cycle). The stance phase starts with heel contact and ends at toe-off. Hence the average sensor reading of the FM-PLS started to increase at the beginning of the cycle (i.e., heel contact), which is clearly visible from the data shown in Figure 16. A threshold of 1890 mV was empirically selected based on the data for the heel strike detection, and the prosthesis controller switches to the stance phase when the sensor reading is greater than the threshold. Conversely, the sensor reading drops immediately after the toe-off, which is also obvious from the result. The threshold for the toe-off was selected as 1895 mV, below which the controller switches to the swing phase. Both of these thresholds were successfully used by the controller for the seamless switching between stance and swing phases without any misdetection during the prosthesis walking.

## 5. Discussion

A novel FM-PLS was designed, developed, and tested in this study. The sensor is compact and lightweight. The height of the device is only 25 mm, adding very small build height to the prosthesis when attached. The unique functioning mechanism (magnetic sensing of millimeter-scale deflection of an elastic element) makes the device more robust against interferences and drifting. The sensor does not require highly accurate placement of magnets as the sensor can easily tolerate misalignment.

As shown in the benchtop testing results in Figure 11, the FM-PLS is sensitive at light load, which makes it competitive for prosthesis control applications with its capability of reliably detecting the initiation and ending of ground contact. Although the sensor is sensitive at light load, it can still withstand heavy load (5000 N axial force) without structural failure, supported by the FEA analysis shown in Figure 7. The benchtop testing results validated the theoretical analysis by demonstrating similar shapes of the axial load vs. sensor response curve and the axial load vs. magnetic flux curve, as shown in Figure 13.

The prosthesis testing data shows that this device can reliably detect the stance phase and swing phase. The sensor signal started to rise immediately after the heel contact and drops after the toe-off as shown in Figure 15. Additionally, sensor signals were used by the prosthesis finite-state controller to successfully trigger the stance and swing phase, which shows great potential of this sensor for real-time powered prosthesis control.

Different from the traditional strain gauge-based force sensors, the FM-PLS load sensing is based on the millimeter-scale deflection of an elastic element, which is several orders of magnitude greater than the strain in a typical load-bearing structure. As such, the FM-PLS is expected to provide stronger signals that are more robust against interferences and drifting. Due to the limitation of experimental conditions, the authors were not able to conduct long-term experiments to validate such a hypothesis. Such experiments are planned as an important part of the future work for further validating and improving the sensor performance.

When compared against similar millimeter-scale deflection-based load sensing approaches (e.g., [19]), the unique design of the FM-PLS incorporates a dedicated elastic element to measure the load (axial force and bending moment) through its deflection, which is easy and less expensive to manufacture since no high-accuracy machining is needed. The cost of the mechanical and electrical components was ∼$1000, which may further decrease in future mass production. As such, it may become a building block for the future of low-cost, lower-limb prostheses to benefit more amputees in their daily life. On the other hand, because of this dedicated elastic element, the FM-PLS sensor is also slightly bigger and heavier than other state-of-the-art prosthetic load sensors (e.g., [19]). With respect to the sensing performance, the RMSE calculated from the FM-PLS experimental data was 28.59 N and the R-value was 0.9802, while the corresponding values in [19] were 60 N and 0.990, respectively. As such, the FM-PLS performance, based on the available experimental data, is comparable to similar sensors, and a more detailed comparison can be conducted when more data become available.

Finally, future work is planned to further validate and improve the performance of the proposed FM-PLS sensor. For the validation of the FM-PLS sensor performance, the authors plan to test the sensor with more sophisticated commercial testing machines to obtain more data on the sensing accuracy, repeatability, and sensor durability. The authors also plan to incorporate the sensor into a robotic lower-limb prosthesis to test its performance in the desired use conditions and characterize the sensor’s reliability and the signals robustness against interferences and drifting. To further improve the sensor performance, structural design may be further improved to reduce the sensor dimensions and improve the sensitivity. Further, wireless communication interface (such as Bluetooth) and an onboard battery may also be introduced to convert the FM-PLS into a wireless sensor, enabling it to serve new applications beyond prosthesis control (gait data collection, activity recognition, etc.).

## 6. Conclusions

This article presents a novel force/torque sensor named FM-PLS. Unlike existing strain gauge-based loadcells, the FM-PLS is based on the measurement of millimeter-scale deflection of an elastic element. The much more significant deflection (compared with the strain of a typical load-bearing structure) may generate stronger sensor signals more robust against interferences and drifting. The design incorporates unique, curved supporting blocks, which modulate the deflection of the elastic member and thus make the measurement sensitive at light load while withstanding heavy load without damage. The FM-PLS prototype is compact and lightweight. With its standard mechanical interface and embedded electronic circuits, the sensor can be easily incorporated into a smart or robotic prosthesis and supply the important structural load information to the prosthesis control system. For the analysis of the sensing mechanism, a two-stage transduction model was established to describe the relationship between the applied force/moment and the output signals. The benchtop testing results match well with the model-driven predictions. To validate its performance, experiments were conducted on a custom testing setup, and the sensor signals displayed a good match to the readings of an off-the-shelf loadcell, as demonstrated by the R-square value of 0.9802 and the RMSE of 28.59 N. Additionally, the sensor was validated through robotic prosthesis walking experiments, and the results showed that the proposed FM-PLS can reliably detect important gait events such as heel strike and toe-off, providing important real-time information to support the intelligent motion control of smart prostheses.

## Figures and Tables

**Table 1:** The material properties of the elastic member.

Orthotropic Elasticity	
Young’s modulus X direction	5.8 × 10^6^ psi
Young’s modulus Y direction	1.5 × 10^6^ psi
Young’s modulus Z direction	1.5 × 10^6^ psi
Poisson’s Ratio XY	0.3
Poisson’s Ratio YZ	0.3
Poisson’s Ratio XZ	0.3
Shear Modulus XY	6 × 10^5^ psi
Shear Modulus YZ	4.2 × 10^5^ psi
Shear Modulus XZ	6 × 10^5^ psi

## Data Availability

The data that support the findings of this study are available from the corresponding author, XS, upon reasonable request.
